# Haemodynamic response to exercise in patients with hypertrophic cardiomyopathy without left ventricular outflow tract obstruction

**DOI:** 10.1136/heartjnl-2025-326193

**Published:** 2025-12-07

**Authors:** Helga Lillian Gudmundsdottir, Anna Axelsson Raja, Kasper Rossing, Hanne Rasmusen, Martin Snoer, Lars Juul Andersen, Rikke Gottlieb, Alex Hørby Christensen, Henning Bundgaard, Emil Wolsk, Christian Hassager, Finn Gustafsson, Jens Jakob Thune

**Affiliations:** 1 Cardiology, Bispebjerg Hospital, Copenhagen, Denmark; 2 Rigshospitalet, Kobenhavn, Denmark; 3 Department of Cardiology, Rigshospitalet, Copenhagen, Denmark; 4 Cardiac, Bispebjerg Hospital, Kobenhavn, Denmark; 5 Cardiology, Sjællands Universitetshospital Roskilde, Roskilde, Denmark; 6 Department of Cardiology, Copenhagen University Hospital Rigshospitalet, Copenhagen, Denmark; 7 Herlev Hospital, Herlev, Denmark; 8 Cardiology, The University Hospital of Copenhagen, Copenhagen, Denmark; 9 Department of Cardiology, Bispebjerg Hospital, University of Copenhagen, Copenhagen, Denmark

**Keywords:** Cardiomyopathy, Hypertrophic

## Abstract

**Background:**

Dyspnoea and reduced exercise capacity are common features in hypertrophic cardiomyopathy (HCM) without left ventricular outflow tract (LVOT) obstruction, yet the haemodynamic mechanisms contributing to these symptoms are poorly described. This study aimed to characterise the haemodynamic response to exercise in patients with HCM without LVOT obstruction.

**Methods:**

In this observational study, 121 subjects underwent right heart catheterisation with haemodynamic measurements during rest and supine incremental exercise: 59 patients with HCM without LVOT obstruction (58±12 years) and 62 healthy controls (49±17 years). Left ventricular (LV) filling pressure was assessed as pulmonary capillary wedge pressure (PCWP) and compared with diagnostic thresholds (rest: <15 mm Hg; exercise: <25 mm Hg). Group comparisons were performed using Student’s t-tests or Wilcoxon rank-sum tests, as appropriate

**Results:**

At rest, patients had higher PCWP than controls (14±6 vs 9±2 mm Hg, p<0.001), while other haemodynamic parameters were similar. Already at mild exercise (25 W), patients showed elevated PCWP (27±7 vs 16±7 mm Hg, p<0.001), mean arterial pressure (100±18 vs 93±13 mm Hg, p=0.02), central venous pressure (12±6 vs 9±4 mm Hg, p<0.001) and mean pulmonary artery pressure (38±11 vs 26±8 mm Hg, p<0.001). At peak exercise, patients exhibited higher PCWP (32±8 vs 18±8 mm Hg, p<0.001), with lower cardiac index (6.4±1.8 vs 8.9±1.4 L/min/m², p<0.001), heart rate (114±21 vs 132±19 bpm, p<0.001) and arteriovenous oxygen difference (58±13 vs 64±6%, p=0.004). Peak exercise capacity (119±50 vs 149±40 W, p<0.001) and peak oxygen consumption (21±7 vs 35±8 mL/kg/min, p<0.001) were lower in patients. Abnormal PCWP was found in 68% of patients at mild exercise and 85% at peak, compared with 6% of controls at mild exercise and 24% at peak. PCWP at mild exercise was inversely correlated with peak oxygen consumption in patients (r=−0.61; 95% CI −0.7 to –0.4; p<0.001).

**Conclusion:**

Patients with HCM without LVOT obstruction exhibited significant haemodynamic impairments during exercise. Abnormally elevated LV filling pressures were present even at low workloads and were correlated with reduced exercise capacity.

**WHAT IS ALREADY KNOWN ON THIS TOPIC**

- Patients with hypertrophic cardiomyopathy (HCM) without left ventricular outflow tract (LVOT) obstruction often experience symptoms like dyspnoea and exertional limitations, which may persist despite currently available treatments. Managing these patients presents a major clinical challenge.

**WHAT THIS STUDY ADDS**

- This study reveals that patients with HCM without LVOT obstruction exhibit significant haemodynamic impairments during exercise, primarily marked by pathologically elevated left ventricular filling pressures even at low workloads. These impairments were closely associated with reduced exercise capacity.

**HOW THIS STUDY MIGHT AFFECT RESEARCH, PRACTICE OR POLICY**

- These findings highlight critical mechanisms contributing to exercise intolerance in patients with HCM without LVOT obstruction. A better understanding of the haemodynamic response to exercise in these patients could inform the development of more effective treatments and improve clinical outcomes for this condition.

## Introduction

Hypertrophic cardiomyopathy (HCM) is a global health burden affecting millions worldwide.^1 2^ Exertional dyspnoea and exercise intolerance, characterised by reduced peak oxygen consumption (peak VO_2_), are hallmark symptoms in HCM. These symptoms are present in patients both with and without left ventricular outflow tract (LVOT) obstruction.^3^ However, the currently available pharmacological options for the one-third of patients with HCM without LVOT obstruction do not effectively improve exercise capacity, and the primary pathophysiology in this population remains poorly understood.^3–8^ This unmet need highlights the necessity of a deeper understanding of the underlying disease mechanisms.

While the pathophysiological features of symptoms and exercise limitations in HCM are likely multifactorial, impaired diastolic function is believed to be a predominant factor.^5 9^ However, the precise haemodynamic mechanisms contributing to exercise intolerance in patients with HCM without LVOT obstruction remain elusive.^10^ Prior studies within the field have used diverse techniques to explore the mechanisms underlying exercise intolerance, including invasive assessments, but few have directly examined exercise haemodynamics using gold-standard right heart catheterisation (RHC).^11–13^ Importantly, none of these studies have addressed exercise haemodynamics in the population of patients with HCM without LVOT obstruction.

We have previously reported the effects of an exercise training programme on haemodynamic responses to exercise in patients with HCM without LVOT obstruction.^14^ In this study, we provide a more detailed analysis of invasively determined haemodynamic responses in these patients, compare them to those of healthy individuals and examine their correlation with peak oxygen uptake.

## Methods

### Study design

This study used baseline data from a clinical trial in patients with HCM without LVOT obstruction (WEDGE-HCM: Will Elevated Left Ventricle Filling Pressure Decrease by a Group Exercise Program in Patients With Hypertrophic Cardiomyopathy?)^14^ and an observational study of healthy individuals (HemReX).^15^ The primary findings of these studies have been published previously.^14 15^ The two study samples were both recruited from institutions in Eastern Denmark, without differences in geographical location or access to healthcare services. All participants provided oral and written informed consent before enrolment. The protocols were published on ClinicalTrials.gov before enrolment of participants (NCT03537183, NCT01974557). Data supporting the findings of this study can be obtained from the corresponding author on reasonable request. The design, conduct and dissemination of this research were carried out without direct input from patients or public representatives.

### Hypertrophic cardiomyopathy population

Patients with HCM without LVOT obstruction were enrolled in a randomised clinical trial designed to assess the haemodynamic effects of a moderate-intensity training programme. Enrolment took place between 2019 and 2022. All measurements included in the current study were obtained before randomisation. HCM was defined as a maximal wall thickness ≥15 mm in the absence of abnormal loading conditions causing the observed phenotype or a maximal wall thickness ≥13 mm in patients with at least one first-degree relative and a definite or likely disease-causing genetic mutation. The cohort included adult patients (>18 years of age) with a confirmed diagnosis of HCM without LVOT obstruction (defined as LVOT gradients <30 mm Hg at rest or induced by Valsalva manoeuvre, or <50 mm Hg induced by exercise). The main exclusion criteria were: no history of exercise-induced syncope or ventricular arrhythmias, severe angina pectoris (Canadian Cardiovascular Society class III–IV), haemodynamically severe valvular disorders, septal reduction therapy within 3 months, severe hypertension (defined as systolic blood pressure >200 mm Hg or diastolic blood pressure >110 mm Hg), inability to exercise due to orthopaedic or other non-cardiovascular limitations, pregnancy and participation in dedicated moderate or high-intensity exercise exceeding 1 hour weekly. Additional information on the trial design has been published.^14^

### Healthy population

The healthy comparison population consisted of adults meeting the following criteria: no history of acute or chronic cardiac or pulmonary disease; normal echocardiogram results without structural cardiac changes, reduced LVEF or significant valvular disease; normal spirometry for their age; routine blood chemistry, including N-terminal pro-B-type natriuretic peptide levels within the normal reference range; body mass index (BMI) between 20 and 30 kg/m^2^, and a standardised cardiopulmonary exercise test (CPET) with ECG showing no pathological findings. Further details on the study design and setting have been reported.^15^

### Outcomes and assessments

All patients underwent RHC with measurements at rest and during exercise, standard transthoracic echocardiography (TTE) and CPET. Genetic testing (in patients) and blood samples (all participants) were analysed according to routine clinical practice. All medications remained unchanged before any testing in patients. Patients and healthy subjects were examined with the same equipment and exercise protocol. We compared the results of invasive haemodynamic parameters at rest and during exercise in patients to those of healthy subjects. Additionally, we evaluated the pulmonary capillary wedge pressure (PCWP) of patients with HCM against established diagnostic criteria for abnormal PCWP at rest and during exercise. In the absence of universally agreed-upon criteria for abnormal exercise responses in HCM, we applied diagnostic thresholds established in heart failure with preserved ejection fraction (HFpEF) (PCWP ≥15 mm Hg at rest and ≥25 mm Hg during exercise), as supported by prior research and European Society of Cardiology guidelines.^16 17^ Lastly, we assessed the relationship between peak VO_2_ and invasive haemodynamic variables in patients with HCM.

### Cardiopulmonary exercise testing

A standard CPET was conducted upright using a cycle ergometer to determine peak VO_2_, with breath-by-breath gas analysis and continuous heart rate and blood pressure monitoring. The participants followed a protocol that included continuous workload increments until the attainment of peak VO_2_, aiming at a respiratory exchange ratio ≥1.1.^18^ Postexercise LVOT gradients of patients were assessed with echocardiography within 1 min of reaching peak VO_2_.

### Right heart catheterisation protocol

The RHC protocol was performed 1–2 weeks following the CPET. A standard 7.5-F triple lumen Swan-Ganz catheter (Edwards Lifesciences, Irvine, California, USA) was inserted under local anaesthesia into the internal jugular vein and advanced to reach the pulmonary artery. All measurements were performed in the supine position: first in a resting position lying flat, then on an ergometer bike (e-bike; GE Healthcare, Fairfield, Connecticut, USA) with legs raised perpendicular to the torso (leg-raise), followed by supine exercise measurements (participant positioning is illustrated in online supplemental figure 1). After completing resting measurements, participants exercised at 60 revolutions per minute. Measurements were made 1 min into each interval. The measurement protocols differed slightly between the patients with HCM and healthy controls. In the HCM group, haemodynamics were measured at rest, during leg raise, at mild exercise (25 W) and maximal exercise workload (75% of the peak VO₂ attained during upright CPET). Additionally, PCWP and mean pulmonary artery pressure (mPAP) were measured at workloads corresponding to 25% and 50% of peak VO₂. In the healthy control group, measurements were taken at rest, during leg raise, and at 25%, 50% and 75% of peak VO₂. A subset of healthy participants (n=46) also underwent measurements at 25 W.

The following variables were obtained during the procedure: mPAP, central venous pressure (CVP), mean arterial pressure (MAP) and PCWP. At rest, PCWP was recorded at end-expiration. During exercise, PCWP was averaged over a 10 s interval to account for respiratory variability. PAP and right atrial pressure (CVP) were measured via the Swan-Ganz catheter positioned in the pulmonary artery and right atrium, respectively. Characteristic pressure waveforms were displayed continuously on a calibrated haemodynamic monitoring system. Central venous blood samples were drawn from the tip of the indwelling pulmonary artery catheter for analyses of lactate and mixed venous oxygen saturation (SvO_2_) concentrations. Cardiac output (CO) was measured using thermodilution as the average of three measurements with <10% variance at each stage, including during exercise. To ensure sufficient time, CO measurements were initiated as the first assessment promptly, 1 min into each workload stage, as they were the most time-consuming. All other haemodynamic parameters were recorded continuously and could be captured concurrently or immediately thereafter without delaying the protocol. Heart rate, non-invasive peripheral oxygen saturation and non-invasive systolic and diastolic blood pressure were monitored continuously during the procedure. The following derived variables were obtained from the source data: pulmonary vascular resistance (PVR) in Wood units was determined by the formula: (mPAP − PCWP)/CO; workload‐corrected PCWP normalised to body weight (PCWL) was assessed using the formula: PCWP/(body weight×Watts); Systemic vascular resistance as 80 × (MAP – CVP)/CO; a surrogate for the arteriovenous oxygen (AV O2) difference was estimated as peripheral oxygen saturation (index finger) – mixed SvO_₂_; body surface area (BSA) was derived using the Du Bois’ formula; cardiac index (CI) was calculated by indexing CO to BSA. To account for age-related differences during maximum workload levels, heart rate was expressed as a percentage of predicted heart rate, calculated using the standard formula: 220 minus age.

### Standard resting transthoracic echocardiography

A standard resting TTE was performed according to echocardiographic core laboratory standards, and analyses were performed by standardised criteria.^19 20^

### Statistical analysis

Data was summarised using mean±SD for normally distributed variables or median (IQR) for non-normally distributed continuous variables and numbers and percentages for categorical variables. To assess differences in continuous variables between groups, the Student’s t-test and Wilcoxon rank-sum test were employed for normally and non-normally distributed variables, respectively. Group differences in categorical variables were analysed using the χ² test. Univariate analysis using Pearson or Spearman correlation coefficients was performed as appropriate for correlation analyses. Haemodynamic variables with p<0.1 in univariable analysis were included in a multivariable regression model using backward elimination (retention threshold: p<0.1) to allow for exploratory variable selection. Age, sex, BMI and beta-blocker usage were added to the model. PCWP was used in the statistical models as there was collinearity between mPAP and PCWP, as well as between PCWL and PCWP. Due to the lack of direct age-matching, a sensitivity analysis using least squares means was conducted to adjust for age across groups, providing age-adjusted means to account for potential confounding by age differences within the sample. Exploratory correlation analyses were performed between resting echocardiographic parameters (LV wall thickness, E/e′ and left atrial volume index (LAVi)) and PCWP and CO at rest and during exercise. Missing data were handled using complete-case analysis. Only participants with non-missing values for the variables of interest were included in each respective analysis. The overall proportion of missing data was minimal. No corrections were made for multiple testing, as the primary analyses were based on predefined between-group comparisons at key stages of exercise, and statistical tests were repeated a limited number of times for physiologically relevant parameters. A p value of <0.05 was considered statistically significant. Analyses were performed using R Statistical Software (V.4.3.1; R Core Team 2023).

## Results

### Clinical characteristics

A total of 121 participants were included in the current study. The clinical characteristics of the study populations are summarised in table 1. Baseline characteristics of participants and results from the comparison population have been presented in previous separate publications but are included in the tables here for ease of reference^14 15^. Approximately half of the patients (n=33) were on beta-blockers, and 7% (n=4) used calcium channel blockers. Genetic testing (available for 95%) showed pathogenic or likely pathogenic mutations in 42% (n=25). Only 12% of patients met predicted peak VO₂ values per the Wasserman-Hansen equation.^21^ 10 patients (17%) had previously undergone septal reduction therapy. All were non-obstructive at rest and during exercise at the time of assessment.

**Table 1:** Clinical characteristics by groups

	HCM (n=59)	Controls (n=62)
Age (years)*	58 (12)	49 (17)
Female sex*	16 (27)	32 (52)
White	58 (98)	62 (100)
BMI, kg/m^2^*	28 (7)	24 (3)
Comorbid conditions		
Hypertension	25 (42)	0
Atrial fibrillation	15 (25)	0
Coronary artery disease	9 (15)	0
Diabetes mellitus	4 (7)	0
Stroke	4 (7)	0
Lung disease	2 (3)	0
NYHA class		
I	13 (22)	N/A
II–III	46 (78)	N/A
NT-pro-BNP, pmol/L*	51 (83)	9 (5)
Heart rate, bpm	64 (12)	63 (10)
SBP, mm Hg	134 (18)	132 (20)
DBP, mm Hg*	81 (14)	76 (14)
Peak VO_2_, mL/kg/min*	21 (7)	35 (8)
% predicted peak VO_2_, %*	76 (21)	114 (23)
Echocardiographic imaging at rest		
LAV index, mL/m^2^*	33 (8)	24 (5)
LVEF, %	59 (9)	62 (7)
E/e’	9 (5)	8 (2)
E/A	1.3 (0.6)	1.3 (0.7)

Values are mean (SD), median (IQR) and numbers (%). The baseline characteristics of the study cohorts have been previously reported. P values were derived using Student’s t-test or Wilcoxon rank-sum test for continuous variables and χ² or Fisher’s exact test for categorical variables.

* p<0.05 for the difference between groups.

BMI, body mass index; bpm, beats per minute; DBP, diastolic blood pressure; HCM, hypertrophic cardiomyopathy; LAV index, left atrial volume index; LVEF, left ventricular ejection fraction; N/A, not available; NT-pro-BNP, N-terminal pro-B-type natriuretic peptide; NYHA, New York Heart Association; SBP, systolic blood pressure; VO_2_, oxygen consumption.

### Resting haemodynamics

At rest, 58% (n=34) of patients compared with 98% (n=61) in the control group exhibited LV filling pressures <15 mm Hg. LV filling and pulmonary arterial pressures were significantly elevated in patients compared with healthy individuals. Additionally, CVP and PVR were higher in patients. The remaining haemodynamic parameters at rest were comparable to those observed in healthy participants, as detailed in table 2.

**Table 2:** Supine resting and exercise haemodynamics

	Controls (n=62)*	HCM (n=59)	P value
Resting			
PCWP (mm Hg)	9 (8 to 9)	14 (12 to 15)	<0.001
SVR (dyn × s/cm5)	1326 (1246 to 1406)	1264 (1163 to 1364)	0.19
HR (beats/min)	63 (61 to 66)	64 (61 to 67)	0.76
CI (L/min/m2)	2.7 (2.6 to 2.8)	2.7 (2.6 to 2.8)	0.70
a-vO2, %	24 (23 to 25)	28 (26 to 29)	0.62
MAP (mm Hg)	89 (86 to 91)	90 (87 to 93)	0.40
CVP (mm Hg)	5 (5 to 6)	6 (5 to 7)	0.05
Lactate (mmol/L)	0.6 (0.6 to 0.7)	1.0 (0.9 to 1.2)	<0.001
mPAP (mm Hg)	14 (14 to 15)	21 (18 to 22)	<0.001
PVR (Wood units)	1.1 (1.0 to 1.2)	1.6 (1.3 to 1.9)	0.03
Leg-raise			
PCWP (mm Hg)	14 (13 to 15)	18 (16 to 19)	<0.001
HR (beats/min)	70 (67 to 73)	64 (61 to 67)	0.008
MAP (mm Hg)	89 (85 to 92)	96 (92 to 100)	0.009
CVP (mm Hg)	9 (8 to 9)	8 (7 to 10)	0.72
mPAP (mm Hg)	21 (19 to 22)	26 (24 to 29)	<0.001
Mild exercise (25 watts)†			
PCWP (mm Hg)	16 (14 to 18)	27 (25 to 29)	<0.001
SVR (dyn × s/cm5)	758 (704 to 812)	813 (748 to 879)	0.42
HR (beats/min)	85 (81 to 89)	86 (82 to 89)	0.69
CI (L/min/m2)	4.8 (4.5 to 5.1)	4.4 (4.1 to 4.7)	0.05
a-vO2, %	43 (41 to 45)	50 (47 to 53)	0.0001
MAP (mm Hg)	93 (89 to 98)	100 (95 to 105)	0.02
CVP (mm Hg)	9 (7 to 11)	12 (11 to 14)	0.001
Lactate (mmol/L)	0.9 (0.8 to 1.1)	1.9 (1.7 to 2.2)	<0.001
mPAP (mm Hg)	26 (23 to 28)	38 (35 to 40)	<0.001
PVR (Wood units)	1.1 (1.0 to 1.2)	1.3 (1.0 to 1.6)	0.42
PCWP/CI	3.3 (2.9 to 3.7)	7.0 (6.3 to 7.7)	<0.001
25% of workload at peak VO_2_			
PCWP (mm Hg)	16 (15 to 19)	28 (26 to 30)	<0.001
mPAP (mm Hg)	25 (24 to 27)	39 (36 to 41)	<0.001
Workload (W)	31 (29 to 34)	30 (26 to 33)	0.16
50% of workload at peak VO_2_			
PCWP (mm Hg)	17 (16 to 19)	31 (28 to 32)	<0.001
mPAP (mm Hg)	30 (28 to 32)	42 (40 to 46)	<0.001
Workload (W)	89 (82 to 96)	59 (53 to 66)	<0.001
Maximal exercise (75% of workload at peak VO_2_)			
PCWP (mm Hg)	18 (16 to 20)	32 (30 to 34)	<0.001
SVR (dyn × s/cm5)	492 (451 to 534)	609 (555 to 663)	<0.001
HR (beats/min)	132 (126 to 137)	114 (108 to 119)	<0.001
HR (% predicted)	78 (74 to 81)	70 (66 to 74)	<0.001
CI (L/min/m2)	8.9 (8.6 to 9.3)	6.4 (6.0 to 6.9)	<0.001
a-vO2, %	64 (62 to 65)	58 (55 to 62)	0.004
MAP (mm Hg)	107 (102 to 111)	109 (103 to 115)	0.46
CVP (mm Hg)	9 (8 to 10)	14 (12 to 16)	<0.001
Lactate (mmol/L)	5.9 (5.2 to 6.6)	6.0 (5.3 to 6.7)	0.84
mPAP (mm Hg)	32 (30 to 34)	45 (42 to 48)	<0.001
PVR (Wood units)	0.9 (0.8 to 1.0)	1.1 (0.8 to 1.4)	0.33
PCWP/CI	2.2 (1.8 to 2.4)	5.6 (4.9 to 6.3)	<0.001
PCWL (mm Hg/W/kg)	35 (31 to 39)	54 (49 to 59)	<0.001
Workload (W)	149 (139 to 159)	119 (106 to 132)	<0.001

Values are mean, 95% CIs. P values were derived using Student’s t-test or Wilcoxon rank-sum test for continuous variables and χ² or Fisher’s exact test for categorical variables.

* Data from the healthy cohort have been published previously but are shown here for ease of reference.

† N=46 for controls at 25 watts.

a-vO2, arteriovenous oxygen difference; CI, cardiac index; CVP, central venous pressure; HCM, hypertrophic cardiomyopathy; HR, heart rate; MAP, mean arterial pressure; mPAP, mean pulmonary arterial pressure; PCWL, workload corrected pulmonary capillary wedge pressure; PCWP, pulmonary capillary wedge pressure; PVR, pulmonary vascular resistance; SVR, systemic vascular resistance.

### Haemodynamic response to exercise

Table 2 details the progression of haemodynamic parameters during exercise.

Left-sided filling pressures were significantly higher in patients than in healthy subjects at all levels of exercise (figure 1). PCWP in patients increased from 13.7±6.7 mm Hg at rest to 32.2±7.5 mm Hg at peak exercise, while for healthy participants, it was within threshold values at all stages of exercise. The largest increase in PCWP in patients occurred at the onset of exercise, with a twofold increase from rest to mild exercise (25 W), in contrast to a 1.2-fold increase from 25 W to peak exercise (p<0.001). Abnormally elevated LV filling pressures (PCWP ≥25 mm Hg) in response to exercise were observed in 68% (n=40) of patients during mild exercise. This increased to 75% (n=44) at a workload corresponding to 25% of peak VO_2_, 78% (n=46) at 50% of peak VO_2_ and 85% (n=50) at maximal exercise. In contrast, 6% (n=4) of controls had elevated filling pressures during mild exercise, increasing to 24% (n=15) during maximal exercise.

**Figure 1:**
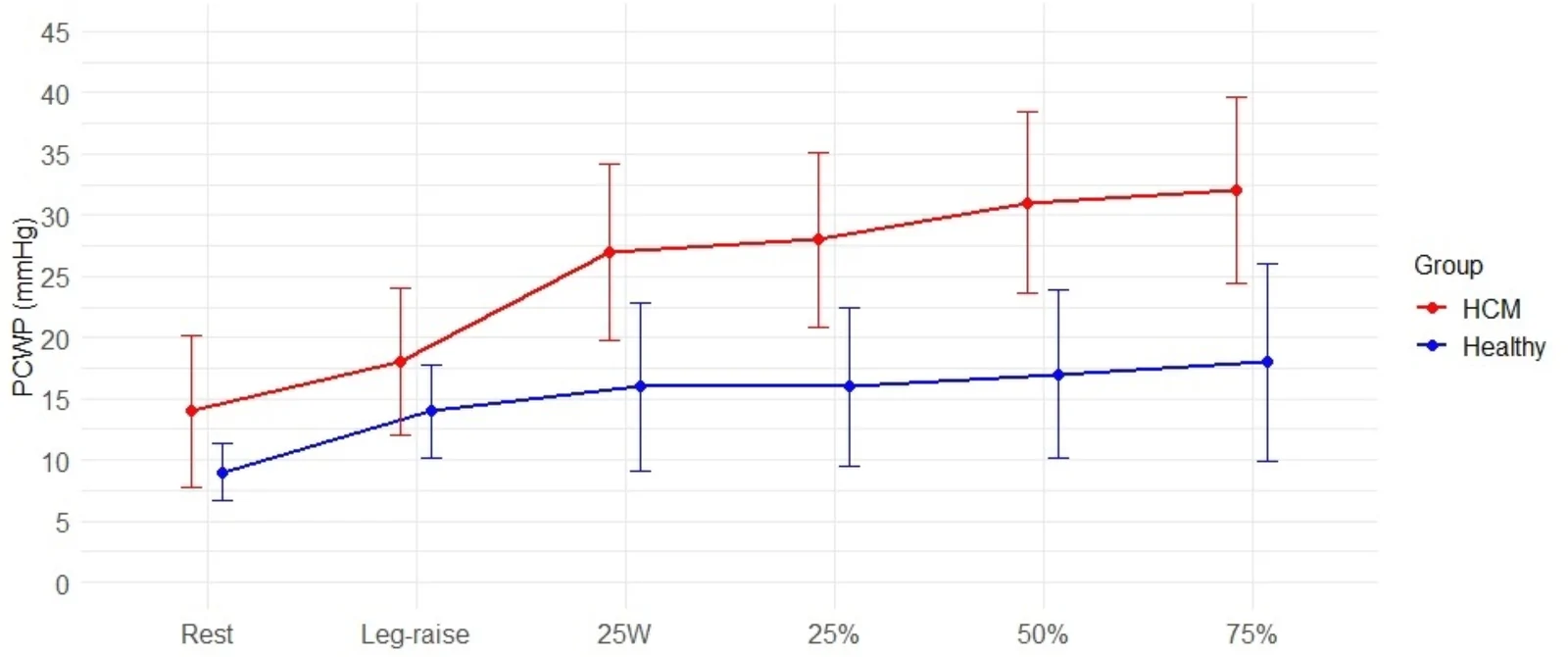
PCWP during rest and exercise. Values are mean PCWP, error bars indicate SD. PCWP was measured at rest and during leg raise, then during exercise at 25 W, at 25%, 50% and 75% of maximal workload. p<0.05 between groups at rest, during leg raise and all levels of exercise. HCM, hypertrophic cardiomyopathy; PCWP, pulmonary capillary wedge pressure.

AV O2 difference was higher in patients compared with controls during mild exercise, but patients showed significantly lower values during maximal exercise than healthy subjects. In patients, the AV O2 difference increased progressively with oxygen demand, rising by a factor of 1.8 from rest to 25 W exercise and by a factor of 2 from rest to peak exercise.

In patients, the CI rose in response to exercise by a factor of 1.6 from rest to mild workload (25 W) and approximately 2.4 from rest to maximal exercise. There was no difference between the groups in CI at mild exercise, but during maximal exercise, CI was significantly lower in patients (p<0.001). Patients not receiving beta-blockers (n=26, 44%) demonstrated a higher CI during maximal exercise compared with those on beta-blockers (7.1±1.2 L/min/m² vs 6.0±2.0 L/min/m², (95% CI 0.3 to 2.0), p=0.013). However, in comparison to healthy subjects, the non-beta-blocker group showed a lower CI during maximal exercise (7.1±1.2 L/min/m² vs 8.9±1.4 L/min/m², (95% CI 1.2 to 2.4), p<0.001).

PCWP/CI values measured during mild exercise showed a significantly higher ratio in patients (7.0 mm Hg/L/min (95% CI 6.3 to 7.7)) compared with healthy individuals (3.3 mm Hg/L/min (95% CI 2.9 to 3.7)), p<0.001. Similarly, during maximal exercise, the PCWP/CI ratio was significantly higher in the patient group (5.6 mm Hg/L/min (95% CI 4.9 to 6.3)) compared with the healthy individuals (2.2 mm Hg/L/min (95% CI 1.8 to 2.4)), p<0.001.

Heart rate increased by 35% from rest to 25 W and 78% from rest to peak exercise in patients, and it did not differ from heart rate in healthy participants measured during mild exercise. However, patients did not increase their heart rate to the extent of healthy subjects in response to maximal exercise, and it was significantly lower at maximal exercise levels compared with healthy individuals. Among patients, those receiving beta-blockers (n=33, 56%) had a lower heart rate during maximal exertion (125±27 bpm) compared with those not on beta-blockers (144±27 bpm) (95% CI 5 to 33), p=0.009. Furthermore, patients not receiving beta-blockers demonstrated a higher heart rate during maximal exercise than healthy controls (144±27 bpm vs 132±19 bpm) (95% CI −24 to −1), p=0.04.

Right atrial pressures increased in both groups with exercise and were significantly higher during exercise in patients compared with healthy individuals. There was a consistent increase in MAP with exercise in both groups, although patients exhibited significantly higher values compared with healthy individuals. Additionally, mPAP increased significantly with exercise in both groups. Systemic and PVRs fell gradually in response to increasing exercise in both groups, and there were no differences in the response to exercise on vascular resistances between the groups. There was a sixfold increase in lactate in response to peak exercise in both groups, indicating maximal effort. The results remained consistent in age-adjusted analyses (online supplemental table 1).

When comparing age-adjusted changes in haemodynamic variables from rest to exercise (online supplemental table 2) between the groups, patients with HCM displayed impaired CI responses, reduced oxygen extraction and elevated PCWP compared with healthy controls.

Compared with healthy controls, PCWP was significantly elevated at rest and during exercise among patients in all age groups (p≤0.01). Figure 2 depicts the differences between patients and healthy individuals at rest and across increasing levels of exercise stratified by age groups. The increase in PCWP was steeper at the onset of exercise in patients compared with healthy controls (online supplemental table 3).

**Figure 2:**
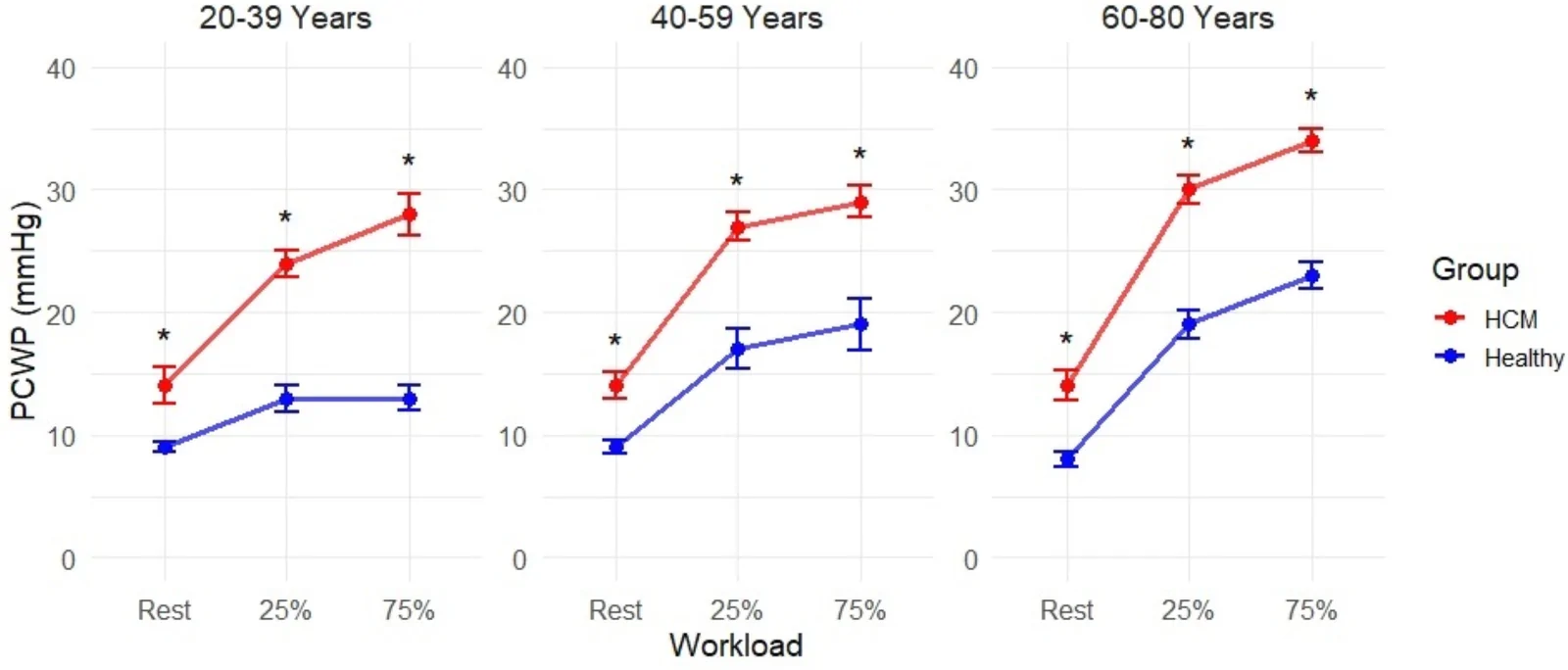
PCWP at rest and during mild exercise stratified by age. Values are mean PCWP from rest to exercise at a workload corresponding to 25% and 75% of maximal workload, stratified by age groups. Error bars indicate SEM. *p<0.05 between groups. Mean age (±SD) for HCM and healthy control subjects within each age group was as follows: 20–39 years—HCM: 34.6±3.7, Controls: 30.0±6.5 (p=0.04); 40–59 years—HCM: 48.7±4.4, Controls: 49.4±4.8 (p=0.66); 60–80 years—HCM: 66.4±3.8, Controls: 68.9±5.5 (p=0.08). HCM, hypertrophic cardiomyopathy; PCWP, pulmonary capillary wedge pressure.

Exploratory analyses (online supplemental table 4) showed that higher E/e′ was weakly associated with elevated PCWP at rest (r=0.26, p=0.04). Greater LAVi correlated with lower CO during mild (r=–0.33, p=0.02) and peak exercise (r=–0.34, p=0.01), as well as with a smaller CO increase from rest to peak (r=–0.31, p=0.03). LV wall thickness was not associated with haemodynamic measures.

### Correlation analyses of haemodynamic parameters and exercise capacity in patients

Within the HCM group, the results of univariate and multivariable regression analyses examining the associations between haemodynamic parameters (at rest and during exercise) and peak VO₂ are presented in table 3. Age, sex, BMI and beta-blocker usage were added to the models. Of haemodynamic variables obtained at rest and 25 W, PCWP was the sole haemodynamic variable independently associated with peak VO_2_. The relationship between peak VO_2_ and PCWP at 25 W (Panel A) and peak VO_2_ and peak CO (Panel B) is illustrated in figure 3. Of measurements obtained at peak exercise, CO and AV O2 difference, but not PCWP, were independently correlated with peak VO_2_.

**Table 3:** Correlations of haemodynamic variables of patients with HCM at rest and during exercise with peak oxygen consumption

Association of haemodynamic variables at rest with peak oxygen consumption				
	Univariable		Multivariable (r^2^=0.56, p<0.001)	
	Coefficient (r) (95% CI)	P value	Coefficient (r) (95% CI)	P value
PCWP (mm Hg)	−0.4 (−0.6 to −0.2)	<0.001	−0.3 (−0.6 to −0.014)	0.04
SVR (dyn × s/cm5)	−0.4 (−0.6 to −0.1)	0.004		
HR (beats/min)	−0.2 (−0.5 to 0.02)	0.06		
CO (L/min)	0.4 (0.1 to 0.6)	0.005		
A-V O2, %	−0.1 (−0.3 to 0.2)	0.46		
MAP (mm Hg)	−0.3 (−0.5 to −0.03)	0.03		
CVP (mm Hg)	−0.4 (−0.6 to −0.2)	<0.001		
mPAP (mm Hg)	−0.6 (−0.8 to −0.5)	<0.001		
PVR (Wood units)	−0.6 (−0.7 to −0.3)	<0.001		

Association of haemodynamic variables during mild exercise (25 W) with peak oxygen consumption				
	Univariable		Multivariable (r^2^=0.63, p<0.001)	
	Coefficient(r) (95% CI)	P value	Coefficient (r) (95% CI)	P value
PCWP (mm Hg)	−0.6 (−0.7 to −0.4)	<0.001	−0.3 (−0.5 to −0.1)	0.002
SVR (dyn × s/cm5)	−0.3 (−0.5 to −0.06)	0.02		
HR (beats/min)	−0.2 (−0.4 to 0.04)	0.099		
CO (L/min)	0.5 (0.2 to 0.6)	<0.001		
A-V O2, %	−0.3 (−0.5 to −0.1)	0.02		
MAP (mm Hg)	0.03 (−0.2 to 0.3)	0.81		
CVP (mm Hg)	−0.5 (−0.7 to −0.3)	<0.001		
mPAP (mm Hg)	−0.7 (−0.8 to −0.6)	<0.001		
PVR (Wood units)	−0.5 (−0.7 to −0.3)	<0.001		
PCWL (mm Hg/W/kg)	−0.38 (−0.6 to −0.1)	0.002		

Association of haemodynamic variables at maximal exercise with peak oxygen consumption				
	Univariable		Multivariable (r^2^=0.69, p<0.001)	
	Coefficient(r) (95% CI)	P value	Coefficient (r) (95% CI)	P value
PCWP (mm Hg)	−0.5 (−0.7 to −0.3)	<0.001		
SVR (dyn × s/cm5)	−0.4 (−0.6 to −0.2)	0.002		
HR (beats/min)	0.5 (0.3 to 0.7)	<0.001		
CO (L/min)	0.6 (0.4 to 0.8)	<0.001	0.6 (0.14 to 1.1)	0.01
A-V O2, %	−0.03 (−0.3 to 0.2)	0.8	0.6 (0.16 to 21.0)	0.046
MAP (mm Hg)	0.04 (−0.2 to 0.3)	0.75		
CVP (mm Hg)	−0.6 (−0.7 to −0.4)	<0.001		
mPAP (mm Hg)	−0.6(−0.7 to −0.4)	<0.001		
PVR (Wood units)	−0.4 (−0.6 to −0.2)	<0.001		
PCWL (mm Hg/W/kg)	0.6 (0.4 to 0.8)	<0.001		

Maximal exercise corresponding to 75% of peak oxygen consumption achieved during an upright cardiopulmonary exercise test. Multivariable models adjusted for age, sex, BMI and beta-blocker usage.

A-V O2, arteriovenous oxygen difference; BMI, body mass index; CO, cardiac output; CVP, central venous pressure; HCM, hypertrophic cardiomyopathy; HR, heart rate; MAP, mean arterial pressure; mPAP, mean pulmonary arterial pressure; PCWL, workload corrected pulmonary capillary wedge pressure; PCWP, pulmonary capillary wedge pressure; PVR, pulmonary vascular resistance; SVR, systemic vascular resistance.

**Figure 3:**
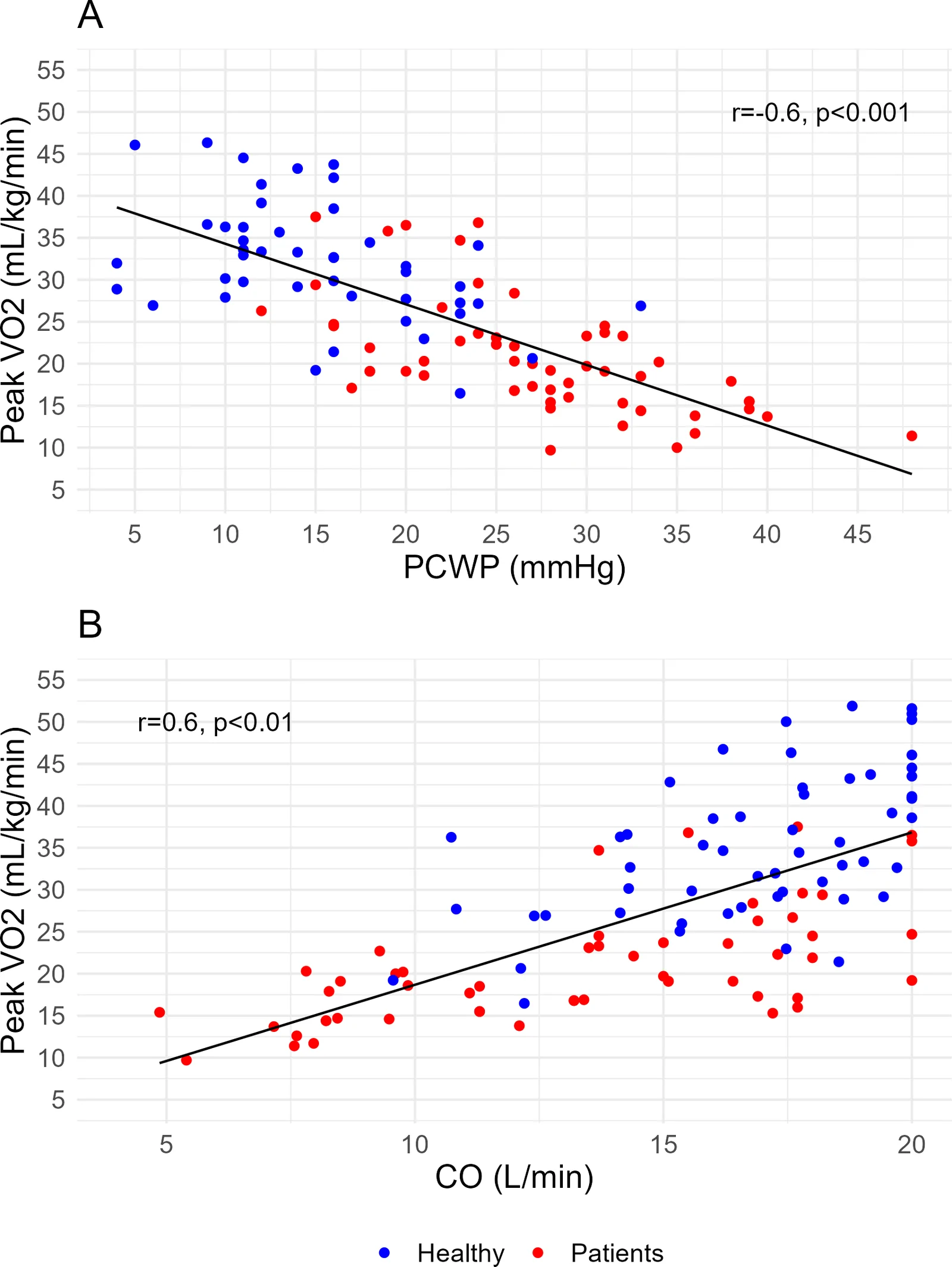
Relationship between peak VO_2_ and haemodynamic parameters. (A) Scatterplot of peak VO_2_ and PCWP during mild exercise (25 W). (B) Scatterplot of peak VO_2_ and CO during maximal exercise. CO, cardiac output; PCWP, pulmonary capillary wedge pressure; peak VO_2_, peak oxygen consumption.

## Discussion

In this study, we compared the haemodynamic response to exercise in a well-defined cohort of patients with HCM without LVOT obstruction with healthy subjects. Exercise was associated with an abnormal and disproportionate increase in PCWP for patients with HCM even at the lowest workloads. Moreover, PCWP at low workload was inversely correlated with peak VO_2_. Compared with healthy controls, PCWP was significantly elevated at rest, during mild and maximal exercise within all age groups in patients with HCM without LVOT obstruction. The significantly higher PCWL in patients with HCM at maximal exercise underscores their haemodynamic impairments relative to healthy controls, regardless of differences in maximal workload levels.

Our findings reveal a notable reduction in exercise capacity among patients with HCM without LVOT obstruction, reinforcing existing evidence that exercise intolerance remains a significant challenge in HCM, irrespective of LVOT pressure gradient. In a systematic review including a meta-analysis of 11.672 patients with HCM (mean age 48±14 years, 66% men, 31.4% with LVOT obstruction at rest), mean peak VO_2_ was 22.3±3.8 mL/kg/min (compared with 20.7±6.6 mL/kg/min in our cohort). Interestingly, peak VO_2_ did not differ significantly between obstructive versus non-obstructive patients.^22^ Similarly, in a study of 1005 patients with HCM (50±14 years, 64% men), only 8% of patients achieved the predicted peak VO_2_ (comparable to the 12% of patients in this present study).^23^

Few studies have measured invasive exercise haemodynamics in HCM, particularly in patients without LVOT obstruction. To our knowledge, this is the first study focusing on this specific patient group.^24^ Two previous studies in patients with HCM (n=23) from 1989 and 1995 found that PCWP did not correlate with peak VO_2_^25 26^. However, the studies included patients with elevated resting LVOT gradients and did not assess LVOT gradients during or postexercise, so results may be confounded by the likely presence of exercise-induced LVOT obstruction. While the clinical significance of this correlation remains exploratory, our findings suggest that submaximal exercise measures may provide additional insight into functional limitation in non-obstructive HCM and could serve as endpoints in future therapeutic trials.

Arena and colleagues demonstrated that CPET results, particularly ventilatory efficiency, correlate with resting haemodynamics in subjects with HCM without LVOT obstruction.^27^ Although their cohort was slightly younger than ours, the reported resting haemodynamic values align with our findings. A recent retrospective analysis of 37 patients with apical HCM (mean age 56±18 years, 43% women) who underwent RHC reported that 78% had elevated LV filling pressures at rest, with a mean PCWP of 20±7 mm Hg.^28^ By comparison, our study found elevated LV filling pressures at rest in 42% of patients, with a mean PCWP of 14±6 mm Hg. The observed differences are likely attributable to the characteristics of the patient cohorts. The patients in our cohort presumably resemble the general HCM population more closely, while those in the retrospective analysis represented a sicker subgroup with unexplained dyspnoea as the most common indication for the RHC procedure. The retrospective analysis had exercise-haemodynamic measurements available for only four patients, limiting the ability to compare exercise responses between studies. In addition, differences in exercise posture (supine vs upright) may have contributed to the discrepancies in haemodynamic findings across the studies discussed.

We observed weak to modest correlations between resting echocardiographic markers and invasive haemodynamics, reflecting the known limitations of E/e′ and LAVi in HCM, consistent with prior studies.^29^ Similarly, LV wall thickness showed no association with haemodynamic measures.^30^

Our findings demonstrate impaired ventricular compliance likely contributing to exercise intolerance in patients with non-obstructive HCM, shown by a marked early rise in LV filling pressures. Mean PCWP differences versus controls were 5 mm Hg at rest, 11 mm Hg during mild and 14 mm Hg at peak exercise. Elevated resting and exercise filling pressures predict worse outcomes in heart failure, and small (5 mm Hg) resting pressure reductions may reduce mortality by up to 30%, highlighting the potential clinical relevance in non-obstructive HCM.^31–33^ PCWP/CI was significantly higher in patients with HCM at mild and maximal exercise, with potential prognostic relevance, as elevated exercise wedge/flow slopes have been associated with reduced exercise capacity and adverse outcomes in HFpEF.^34^

### Limitations

Our study has limitations that warrant consideration. There is a potential for selection bias affecting generalisability as the patients were included in a randomised clinical trial. The age groups were not evenly distributed in the group of patients compared with the cohort of healthy controls used for comparison, and the lack of age and gender matching, along with differences in comorbidities and beta-blocker usage, is a limitation. CO was measured by thermodilution, and the maximum measurable value was 20 L/min. In the population of healthy subjects, medications that could potentially affect the cardiovascular system were paused 48 hours before the invasive tests, whereas patients continued taking their medications. Haemodynamics were evaluated during supine exercise, which may not fully reflect upright physiology. Prior work in HFpEF has shown that PCWP responses can differ between supine and upright exercise, and it is possible that similar posture-related differences could be relevant in HCM.^35^ This factor should be considered when interpreting exercise PCWP as a limiting mechanism. A minority of patients (17%) had previously undergone septal reduction therapy, which may introduce modest heterogeneity, although all met strict criteria for non-obstructive physiology at the time of assessment. Between-group comparisons were not adjusted for multiple comparisons and should be interpreted with appropriate caution. Finally, a statistical analysis examining the impact of sex on haemodynamic function was not feasible due to the predominantly male cohorts.

### Conclusion

Patients with HCM without LVOT obstruction exhibit significant haemodynamic impairments during exercise, marked by disproportionately elevated LV filling pressures even at low workloads. This impaired haemodynamic response correlates with reduced exercise capacity.

## Supplementary material

10.1136/heartjnl-2025-326193 online supplemental file 1

## Data Availability

Data are available upon reasonable request.
