# Supplementary material for: Haemodynamic response to exercise in patients with hypertrophic cardiomyopathy without left ventricular outflow tract obstruction
Source: Heart. 2025 Dec 7;112(19):e326193. doi: 10.1136/heartjnl-2025-326193 (PMC13638292; doi:10.1136/heartjnl-2025-326193)
Supplement: Supplementary file 1 [file heartjnl-112-19-s001.docx]

**SUPPLEMENTARY MATERIAL**

| *Supplementary Table 1*  Age-adjusted supine resting and exercise hemodynamics    Controls  (n=62)* | | HCM  (n=59) | p-value |
| --- | --- | --- | --- |
| Resting | | | |
| PCWP (mmHg) | 9 (7, 9) | 14 (13,15) | <0.001 |
| SVR (dyn x s/cm5) | 1335 (1243, 1428) | 1231 (1138, 1323) | 0.12 |
| HR (beats/min) | 63 (60, 66) | 64 (61, 66) | 0.90 |
| CI (L/min/m2) | 2.7 (2.6, 2.8) | 2.7 (2.6, 2.9) | 0.87 |
| a-v O2, % | 27 (24, 30) | 28 (25, 31) | 0.61 |
| MAP (mmHg) | 90 (87, 93) | 90 (87, 93) | 0.96 |
| CVP (mmHg) | 5 (5, 6) | 6 (6, 7) | 0.05 |
| Lactate (mmol/L) | 0.6 (0.5, 0.7) | 1.0 (0.9, 1.1) | <0.001 |
| mPAP (mmHg) | 15 (13, 16) | 21 (20, 23) | <0.001 |
| PVR (Wood units) | 1.2 (0.9, 1.4) | 1.5 (1.2, 1.7) | 0.12 |
| Mild Exercise (25 watts)* | | | |
| PCWP (mmHg) | 16 (15, 18) | 27 (25, 29) | <0.001 |
| SVR (dyn x s/cm5) | 769 (699, 840) | 807 (750, 864) | 0.41 |
| HR (beats/min) | 85 (81, 90) | 86 (82, 90) | 0.81 |
| CI (L/min/m2) | 4.8 (4.5, 5.2) | 4.4 (4.1, 4.7) | 0.05 |
| a-v O2, % | 44 (41, 46) | 49 (48, 52) | 0.008 |
| MAP (mmHg) | 94 (89, 99) | 100 (96,104) | 0.07 |
| CVP (mmHg) | 9 (8, 11) | 12 (11,13) | 0.007 |
| Lactate (mmol/L) | 1.0 (0.7, 1.2) | 1.9 (1.7, 2.1) | <0.001 |
| mPAP (mmHg) | 27 (24, 29) | 37 (34, 39) | <0.001 |
| PVR (Wood units) | 1.2 (0.9, 1.4) | 1.3 (1.0, 1.5) | 0.56 |
| PCWP/CI | 3.2 (2.6, 3.8) | 5.4 (0.3, 4.8) | <0.001 |
| Maximal Exercise (75% of workload at peak VO_2_) | | | |
| PCWP (mmHg) | 19 (17, 21) | 31 (29, 33) | <0.001 |
| SVR (dyn x s/cm5) | 505 (454, 555) | 589 (542, 635) | 0.02 |
| HR (beats/min) | 130 (125, 135) | 115 (110, 120) | 0.001 |
| CI (L/min/m2) | 8.7 (8.3, 9.1) | 6.7 (6.3, 7.0) | <0.001 |
| a-v O2, % | 64 (61, 67) | 58 (56, 61) | 0.004 |
| MAP (mmHg) | 108 (103, 114) | 108 (103,113) | 0.98 |
| CVP (mmHg) | 10 (8, 11) | 14 (12,15) | 0.006 |
| Lactate (mmol/L) | 5.8 (5.1, 6.5) | 6.2 (5.5, 6.9) | 0.43 |
| mPAP (mmHg) | 34 (32, 37) | 44 (41, 46) | <0.001 |
| PVR (Wood units) | 0.9 (0.7, 1.2) | 1.0 (0.8, 1.3) | 0.55 |
| PCWP/CI | 2.4 (1.9, 2.9) | 5.4 (4.8, 5.9) | <0.001 |
| PCWL (mmHg/W/kg) | 35 (31, 40) | 54 (50, 59) | <0.001 |

Values are mean, 95% confidence intervals. Abbreviations: CI, cardiac index; CVP, central venous pressure; mPAP, mean pulmonary arterial pressure; MAP, mean arterial pressure; PCWL, workload corrected pulmonary capillary wedge pressure; PCWP, pulmonary capillary wedge pressure; PVR, pulmonary vascular resistance; SVR, systemic vascular resistance; a-vO2, arteriovenous oxygen difference; HR, heart rate. *N=46 for controls at 25 watts.

| *Supplementary Table 2*  Age-adjusted differences in hemodynamic changes from rest to exercise | | | | | | |
| --- | --- | --- | --- | --- | --- | --- |
|  | **Controls (n=62)** | | **HCM (n=59)** | **Difference** | **95% CI** | **p-value** |
| Change from rest to mild exercise (25W)* | | | | | | |
| HR (beats/min) | 21 | 22 | | -1,4 | (-6.8, 4.0) | 0.62 |
| MAP (mmHg) | 4 | 10 | | -5,4 | (-11.2, 0.3) | 0.07 |
| avO2 (%) | 18 | 22 | | 4 | (-0.1, 0.0) | 0.04 |
| Lactate (mmol/L) | 0.3 | 0.9 | | -0,6 | (-0.8, -0.3) | <0.001 |
| CVP (mmHg) | 4 | 6 | | -1,7 | (-3.2, -0.2) | 0.03 |
| mPAP (mmHg) | 12 | 16 | | -3,6 | (-5.7, -1.5) | 0.001 |
| PCWP (mmHg) | 8 | 13 | | -4,9 | (-7.1, -2.8) | <0.001 |
| PVR (Wood units) | -0.11 | -0.25 | | 0,1 | (-0.1, 0.4) | 0.30 |
| SVR (dyn x s/cm^5^) | -574 | -440 | | -133,7 | (-267.4, 0.1) | 0.05 |
| CI (L/min/m2) | 2.1 | 1.7 | | 0,4 | (-0.1, 0.8) | 0.02 |
| Change from mild exercise to maximal exercise (75% of workload at peak VO_2_) | | | | | | |
| HR (beats/min) | 45 | 29 | | 16,4 | (8.9, 23.9) | <0.001 |
| MAP (mmHg) | 16 | 9 | | 6,7 | (-2.6, 15.9) | 0.16 |
| avO2 (%) | 21 | 10 | | 10 | (0.1, 0.2) | <0.001 |
| Lactate (mmol/L) | 4.7 | 4.3 | | 0,4 | (-0.7, 1.5) | 0.43 |
| CVP (mmHg) | 1 | 2 | | -0,9 | (-2.0, 0.3) | 0.16 |
| mPAP (mmHg) | 8 | 7 | | 0,6 | (-1.9, 3.0) | 0.66 |
| PCWP (mmHg) | 3 | 5 | | -1,9 | (-4.1, 0.2) | 0.09 |
| PVR (Wood units) | -0.2 | -0.2 | | 0,0 | (-0.2, 0.2) | 0.87 |
| SVR (dyn x s/cm^5^) | -273 | -202 | | -69,8 | (-163, 24) | 0.15 |
| CI (L/min/m^2^) | 3.9 | 2.1 | | 1,8 | (1.3, 2.3) | <0.001 |
| Change from rest to maximal exercise | | | | | | |
| HR (beats/min) | 67 | 51 | | 15,4 | (7.6, 23.2) | <0.001 |
| MAP (mmHg) | 19 | 19 | | 0,3 | (-7.6, 8.1) | 0.95 |
| avO2 (%) | 40 | 30 | | 10 | (0.0, 0.1) | 0.002 |
| Lactate (mmol/L) | 5.2 | 5.1 | | 0,1 | (-1.0, 1.1) | 0.89 |
| CVP (mmHg) | 4.6 | 7.1 | | -2,5 | (-4.3, -0.8) | 0.005 |
| mPAP (mmHg) | 20 | 22 | | -2,8 | (-5.7, 0.1) | 0.06 |
| PCWP (mmHg) | 11 | 17 | | -6,8 | (-9.3, -4.4) | <0.001 |
| PVR (Wood units) | -0.3 | -0.4 | | 0,2 | (-0.1, 0.4) | 0.19 |
| SVR (dyn x s/cm^5^) | -858 | -645 | | -213,2 | (-347.6, -78.9) | 0.002 |
| CI (L/min/m^2^) | 6.0 | 3.9 | | 2,1 | (1.6, 2.7) | <0.001 |

Values are mean, 95% confidence intervals. Abbreviations: CI, cardiac index; CVP, central venous pressure; mPAP, mean pulmonary arterial pressure; MAP, mean arterial pressure; PCWL, workload corrected pulmonary capillary wedge pressure; PCWP, pulmonary capillary wedge pressure; PVR, pulmonary vascular resistance; SVR, systemic vascular resistance; a-vO2, arteriovenous oxygen difference; HR, heart rate. *N=46 for controls at 25 watts.

***Supplementary Table 3***

Increase in PCWP (Rest to 25% of Peak VO_2_) by Age Group

Age Group	Mean Healthy (n)	Mean Patient (n)	p-value

20-39 years	8 (n=20)	10 (n=7)	0.0104

40-59 years	9 (n=22)	13 (n=15)	0.0013

60-80 years	11 (n=20)	16 (n=37)	<0.001

PCWP: pulmonary capillary wedge pressure. Peak VO_2_: peak oxygen consumption.

***Supplementary Table 4. Correlation Between Resting Echocardiographic Parameters and Hemodynamic Responses***

|  | MWT | E/e′ | LAVi |
| --- | --- | --- | --- |
|  | Correlation coefficient (r), (95% CI), p-value | | |
| PCWP at rest (mmHg) | 0.10(-0.2, 0.3), p=0.44 | 0.26(0.007,0.5), p=0.04 | 0.09(-0.2,0.4), p=0.5 |
| PCWP at 25W (mmHg) | -0.22(-0.4,0.06), p=0.09 | 0.17 (-0.1,0.4), p=0.19 | 0.22(-0.05,0.5), p=0.11 |
| PCWP at maximum exercise (mmHg) | -0.14(-0.4,-01), p=0.27 | 0.16 (-0.1,0.4), p=0.24 | 0.17(-0.1,0.4), 0.22 |
| ΔPCWP (mmHg) | -0.10(-0.3,0.2), p=0.46 | -0.04 (-0.3, 0.2), p=0.74 | 0.13 (-0.1,0.4), p=0.34 |
| CO at rest (L/min) | 0.20(-0.003,0.5), p=0.12 | -0.16(-0.4, 0.1), p=0.21 | 0.24(-0.5, 0.01),p=0.08 |
| CO at 25W (L/min) | 0.17(-0.001,0.5), p=0.18 | -0.17(-0.4,0.1), p=0.19 | -0.33(-0.6,-0.2), p=0.02 |
| CO at maximum exercise (L/min) | 0.14(-0.05,0.4), p=0.11 | -0.15(-0.4,0.1), p=0.4 | -0.34(-0.6,-0.2), p=0.01 |
| ΔCO (L/min) | 0.18(-0.09,0.4), p=0.19 | -0.12(-0.4, 0.1), p=0.6 | -0.31 (-0.6, -0.2), p=0.03 |

Abbreviations: MWT = maximum wall thickness; LAVi = left atrial volume index; PCWP = pulmonary capillary wedge pressure; CO = cardiac output; Δ = peak value minus resting value. Maximal exercise corresponded to 75% of peak oxygen consumption achieved during an upright cardiopulmonary exercise test.

***Supplementary Figure 1***

Illustration showing participant positioning at rest (rest and leg-raise) and during exercise.
